# Smart Distance Lab’s art fair, experimental data on social distancing during the COVID-19 pandemic

**DOI:** 10.1038/s41597-021-00971-2

**Published:** 2021-07-15

**Authors:** Charlotte C. Tanis, Nina M. Leach, Sandra J. Geiger, Floor H. Nauta, Fabian Dablander, Frenk van Harreveld, Sanne de Wit, Gerard Kanters, Jop Knoppers, Diederik A. W. Markus, Rick R. M. Bouten, Quinten H. Oostvogel, Meier J. Boersma, Maya V. van der Steenhoven, Denny Borsboom, Tessa F. Blanken

**Affiliations:** 1 University of Amsterdam, Department of Psychology, Amsterdam, 1018 WS the Netherlands; 2 National Institute for Public Health and the Environment (RIVM), Bilthoven, 3721 MA the Netherlands; 3 Centillien B.V., Mierlo, 5731 SG the Netherlands; 4 Focus Technologies B.V., Eindhoven, 5657 EW the Netherlands; 5 Smart Distance Lab, Leiderdorp, 2353 NM the Netherlands

**Keywords:** Human behaviour, Databases

## Abstract

In the absence of a vaccine, social distancing behaviour is pivotal to mitigate COVID-19 virus spread. In this large-scale behavioural experiment, we gathered data during Smart Distance Lab: The Art Fair (*n* = 839) between August 28 and 30, 2020 in Amsterdam, the Netherlands. We varied walking directions (bidirectional, unidirectional, and no directions) and supplementary interventions (face mask and buzzer to alert visitors of 1.5 metres distance). We captured visitors’ movements using cameras, registered their contacts (defined as within 1.5 metres) using wearable sensors, and assessed their attitudes toward COVID-19 as well as their experience during the event using questionnaires. We also registered environmental measures (e.g., humidity). In this paper, we describe this unprecedented, multi-modal experimental data set on social distancing, including psychological, behavioural, and environmental measures. The data set is available on *figshare* and in a MySQL database. It can be used to gain insight into (attitudes toward) behavioural interventions promoting social distancing, to calibrate pedestrian models, and to inform new studies on behavioural interventions.

## Abstract (summary)

**Table:** 

Measurement(s)	Proximity • Movement • Attitudes and beliefs relating to COVID-19 • Indoor environment measures
Technology Type(s)	Ultra-wideband technology • Camera Device • questionnaire • environment sensor
Factor Type(s)	Walking direction • Wearing of face masks • Proximity buzzer
Sample Characteristic - Organism	Homo sapiens
Sample Characteristic - Environment	public exhibition
Sample Characteristic - Location	Kingdom of the Netherlands

Machine-accessible metadata file describing the reported data: 10.6084/m9.figshare.14312180

## Background & Summary

The COVID-19 pandemic severely affected cultural life around the globe, including theatres, exhibitions, museums, and music events^1^. Many countries, including the Netherlands, shut down cultural venues and generally recommended individuals to maintain a distance of 1.5 metres from one another to reduce virus spread^2^. As the pandemic persisted, a key question became whether behavioural interventions could promote social distancing without bringing society to a standstill. Together with Smart Distance Lab, we conducted a field experiment during an art fair in Amsterdam, the Netherlands, to investigate how social distancing can be promoted effectively during large-scale events. Specifically, we implemented several behavioural interventions and assessed their effectiveness in promoting social distancing behaviour. In doing so, we aim to provide insight into how events can be organised safely during a pandemic.

**Fig. 1:**
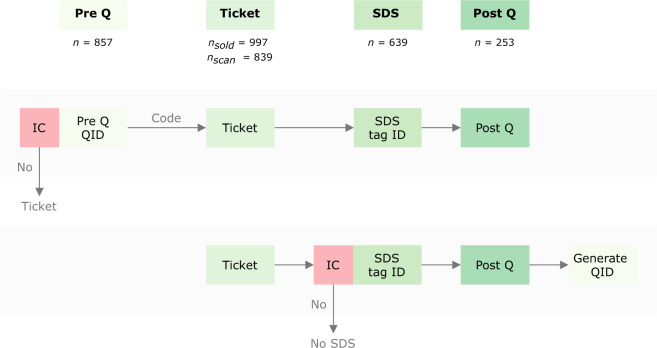
Overview of data collection. The top stream represents visitors who purchased their own ticket. After providing informed consent (IC), visitors completed a pre-questionnaire (Pre Q) which gave access to a code for a ticket. If participants declined informed consent, they received the code immediately. At the art fair, visitors received an SDS before entering and were asked to complete a post-questionnaire (Post Q) when exiting. Visitors in the bottom stream did not complete the pre-questionnaire and were asked to provide informed consent before receiving an SDS. We used the questionnaire IDs (QID) as a unique identifier for participants. For visitors in the bottom stream, we generated QIDs after collecting the data. Camera and indoor environment data were collected throughout the art fair.

The art fair was organised between August 28–30, 2020 and visited by 839 individuals. The study took place between the first and second COVID-19 wave, when about 500 new COVID-19 cases were registered in the Netherlands each day^3^. We implemented a combination of several interventions, including walking directions (bidirectional, unidirectional, no directions) and supplementary interventions (face mask, buzzer via wearable social distancing sensors, none). Before visiting the art fair, visitors completed a questionnaire, which included questions on factors related to adopting social distancing (e.g., perceived risk, norms, and knowledge), see Fig. 1 for a schematic overview. During the visit, we collected distancing data via wearable Social Distancing Sensors (SDSs), movement data via cameras, and indoor environment data such as humidity and temperature. After the visit, we administered an exit questionnaire focusing on experiences with keeping distance during the art fair (e.g., perceived difficulty, adherence, and automaticity). These different data collection modes resulted in a unique data set combining psychological, behavioural, and environmental measures of a large-scale event organised during the COVID-19 pandemic.

The reported data set can, for example, be valuable to provide insight into attitudes and behaviours during the COVID-19 pandemic, to help calibrate pedestrian models, to validate social distancing measurements, and to design subsequent studies investigating behavioural interventions. We initially used these data to investigate how behavioural interventions influence social distancing behaviour, employing Behavioural Contact Networks^4^ (BECONs) that encode which individuals came within 1.5 metres of each other. We subsequently compared the networks across conditions to assess the effectiveness of the interventions in terms of social distancing^5,6^.

## Methods

### Design

**Fig. 2:**
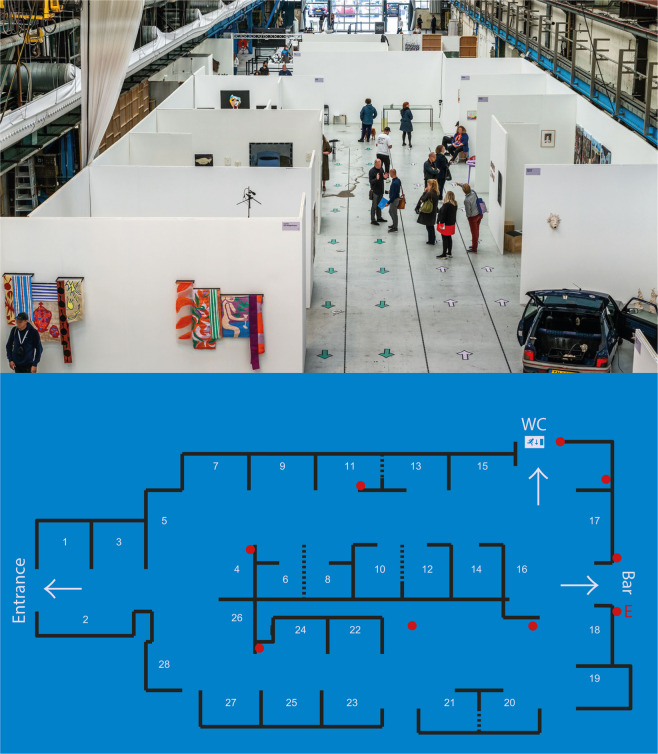
Layout of the art fair. The total area used for the event was divided into three main sections: entrance (500 m2), gallery (1,080 m2), and bar (1,338 m2). Visitors entered on the left side after passing the cloakroom and research desk. The picture was taken on the first day when walking directions were bidirectional. The layout below shows the gallery with the artists’ stands (1–28). Stands were on average 16.68 m2 (*Min* = 13.5 m2, *Max* = 19.5 m2). Due to the layout of the stands, the accessible area in the gallery was smaller than the total floor area. The red dots indicate the fixed places of the nine location badges. The indoor environment was measured at the red letter “E”.

At the art fair, a selection of top graduates of Dutch art academies who finalised their studies in 2019 and 2020 displayed their work in the Kromhouthal in Amsterdam, see Fig. 2. Different time slots during the three-day event allowed for implementing different conditions, as shown in Table 1. The original set-up was a fully crossed design of walking directions (bidirectional, unidirectional, no directions) and supplementary interventions (face mask, buzzer, none). However, we had to adapt this set-up due to unforeseen circumstances. As a result, we could only implement experimental conditions during eight of the 11 available time slots. We also needed to repeat the buzzer condition on day 3 because the buzzer settings differed across conditions (see setting specification in Table 1).

**Table 1:** Descriptives per condition.

Time slot	Day	Start	End	Walking direction	Supplementary intervention	SDS setting	N	Duration (min)
1	28-Aug	08:00	11:30	Bidirectional		No SDS		
2	28-Aug	13:30	15:30	Bidirectional	Facemask	No feedback^a^	130	120
3	28-Aug	15:30	17:30	Bidirectional	None	No feedback^a^	137	120
4	28-Aug	17:30	19:30	Bidirectional	Buzzer	Buzzer after 2 sec	122	120
5	29-Aug	11:00	13:30	Unidirectional		No SDS		
6	29-Aug	13:30	15:30	Unidirectional	None	No feedback^a^	147	120
7	29-Aug	16:00	18:00	Unidirectional	Buzzer	Buzzer immediately, stops after 2 sec	137	120
8	30-Aug	11:00	13:30	No direction		No SDS		
9	30-Aug	13:30	15:30	No direction	Buzzer	Buzzer immediately, stops after 2 sec	123	120
10	30-Aug	15:30	16:30	No direction	Buzzer	Buzzer immediately, persists after 2 sec	146	60
11	30-Aug	17:00	18:00	No direction	None	No feedback^a^	102	60

We implemented experimental conditions in eight time slots (2, 3, 4, 6, 7, 9, 10, and 11). Some of these time slots contained only walking directions (Supplementary intervention = None), while others contained both walking directions and a supplementary intervention. SDSs were not handed out during the remaining time slots (1, 5, and 8). Six experimental conditions lasted two hours, while two lasted one hour. Except in the buzzer conditions, the SDS was covered in a black bag so the flashing light was invisible. Note that the sum of the number of visitors across conditions differs from the reported *n* = 639 visitors who wore an SDS, because some people stayed inside the art fair during multiple conditions.

^a^The SDS requires to always select at least one form of feedback (light, buzzer, sound). In conditions during which the SDS should not provide feedback (face mask, no supplementary intervention), we set the settings to light, and provided black bags to cover the SDS, such that no feedback was received.

In the unidirectional and bidirectional conditions, walking directions were indicated through arrows displayed on the floor. On day 1, arrows were pasted in two directions forming two lanes that guided visitors to walk either clock- or anticlockwise (bidirectional walking condition). On day 2, arrows pointed only in one direction (unidirectional walking condition), while there were no directions on day 3. Within each of these conditions (i.e., during different time slots within each day), we implemented a set of supplementary interventions. For the face mask condition, we handed out face masks to visitors. At the beginning of the buzzer conditions, we operated the SDS such that the sensor would buzz when visitors came within 1.5 metres from one another.

### Sample

In total, 997 tickets were sold for the art fair. During three days, 839 people entered the fair, of which 639 (76.2%) wore an SDS. As shown in Table 1, we measured eight out of 11 time slots. In time slot 5 and 8, 74 and 132 visitors, respectively, already entered the art fair. Some of these visitors had already left before the SDSs were handed out. Thus, the percentage of visitors who wore an SDS during the experimental conditions was higher than the reported 76.2%. We gathered demographic information (*n* = 857) when people obtained a ticket. The average age of this group was 45.2 (*SD* = 16.0, *Min* = 12, *Max* = 82), 54.1% were female, and the majority (83.9%) completed higher education. Figure 1 provides a schematic overview of the different visitor streams.

### Materials

#### Questionnaires

Participants completed two questionnaires: a pre-questionnaire before entering the art fair and a post-questionnaire after attending the event. The pre-questionnaire recorded participants’ demographics (i.e., age, gender, and educational level), email addresses, and whether they had previously been infected with the coronavirus. It included seven items about a potential coronavirus infection: the likelihood of getting infected (0 *very unlikely* to 100 *very likely*), the severity (1 *not serious at all* to 7 *very serious*), the perceived health risk for family and friends, and (separately) for themselves (1 *extremely small* to 7 *extremely large*), as well as worries about getting infected, infecting others, and an overloaded healthcare system (1 *no worries at all* to 7 *a lot of worries*). The pre-questionnaire also contained 16 items about their attitudes and self-reported behaviours regarding the behavioural guidelines, the perceived social norm, and automaticity of keeping distance (four items of the Self-Reported Behavioral Automaticity Index^7^; 1 *completely disagree* to 7 *completely agree*), as well as how participants experience the social distancing rule (1 to 7, *not sensible*-*sensible*, *useless*-*useful*, *not enjoyable*-*enjoyable*, *unfair*-*fair*, *unacceptable*-*acceptable*, *difficult*-*easy*). Finally, participants were asked about their general health (1 *very bad* to 7 *very good*), how they feel about face masks as protection against the coronavirus, and whether it is less important to keep distance when wearing a face mask (1 *completely disagree* to 7 *completely agree*). Online-only Table 1 shows both the original Dutch and translated English questions and response options of the pre-questionnaire.

The post-questionnaire recorded participants’ email addresses to link the two questionnaires. It included 13 items about participants’ social distancing behaviour during the event: whether they tried to maintain distance during the event (1 *completely disagree* to 7 *completely agree*), the experienced difficulty of maintaining distance and determining when someone is within 1.5 metres distance (1 *difficult* to 7 *easy*), their adherence to the 1.5 metre guideline (1 *not at all* to 7 *constantly*), as well as automaticity of distancing, and whether they felt they were constantly reminded of maintaining distance (1 *completely disagree* to 7 *completely agree*). Next, participants were asked to what extent they felt protected against the coronavirus, their stress level during the event, the extent to which they experienced freedom to behave as they wished, to what extent they felt obligated to behave in a certain way (two items), trust in their ability to maintain distance, and pleasure during the event (1 *completely disagree* to 7 *completely agree*). Lastly, they were asked how many units of alcohol they consumed during the art fair and whether they wore a face mask during their visit. Online-only Table 1 shows both the original Dutch and translated English questions and response options of the post-questionnaire.

#### Social distancing sensor

SDSs are wearable electronic devices that use ultra-wideband (UWB) technology to detect the presence of other sensors, and measure distance with an accuracy up to ten centimetres. The SDSs in this study were designed by Focus Technologies B.V. (https://www.findfocus.nl) together with Sentech B.V. (https://www.sentech.nl). Data collection required four types of devices: the SDSs, an access point, a laptop connected to the access point running a control application, and multiple base stations. Each sensor had a unique tag ID and locally stored counts of how often other sensors had been within a pre-specified range, i.e., 1.5 metres. The access point located near the entrance of the gallery area collected these counts when an SDS was within 30 metres (line of sight) and sent the data to a central database. A time stamp was only recorded when the data moved from locally stored on the SDS to the central database and, therefore, does not refer to the time of contact itself. Contacts that occurred near the entrance may have been sent to the database immediately, whereas the majority of SDSs were only close enough to the access point when a data sweep was performed. In addition, the access point updated the settings of the SDSs in case they had been changed via the application. An SDS was (de)activated by placing a sensor on a base station. In our set-up, up to four SDSs could be linked by simultaneously placing them on different base stations to avoid registering contacts between members of the same household. As soon as an SDS was deactivated, any other SDSs that were linked to it were disconnected.

The SDSs were set to register a contact when another SDS was within 1.5 metres. When two SDSs were in contact, they gave at least one of three types of feedback: a flashing light, a buzzing sensation, or a beeping sound. This feedback could either occur immediately or after two seconds of contact. Except in the buzzer conditions, we set the feedback to a flashing light and placed the SDS in a small black bag to avoid visitors being able to see whether they were within 1.5 metres of others. This way, data could be gathered without the flashing light influencing the behaviour of visitors. Visitors wore the SDS on a key cord (lanyard) around their neck. In addition to the SDSs worn by visitors, we positioned nine “location” SDSs at a fixed location inside the fair, see the red dots in Fig. 2. These sensors were only activated outside of the buzzer conditions to prevent inappropriate feedback to visitors when standing close to a location SDS.

#### Cameras

We mounted six optical cameras on trusses at a height of 12 metres. The cameras were configured to record with a resolution of 640 × 480 pixels to ensure that visitors of the art fair could not be recognised from the recorded images. Each camera lens had a field of view of 98° × 55° resulting in a maximum ground coverage of 27.6 × 12.5 metres per camera. Three cameras were mounted above the entrance and covered the entire entrance area. The other three cameras were located above the gallery, covering stands 1 to 17. One of these cameras, covering stands 15 to 17 and the entrance to the toilet, was mounted at an angle of 10° ± 2° to prevent walls from blocking the camera view. The cameras were configured to record at 10 frames per second (FPS) to enable real-time data processing.

#### Indoor environment measures

During the event, the temperature, humidity, and light intensity were continuously monitored using the internal sensors of an Ubibot WS1^8^. The internal temperature sensor had a precision of ±0.3 °C and a range of −20 °C to 60 °C. The internal humidity sensor had a precision of ±3 RH within the range of 10% to 90% relative humidity. The light sensor had a precision of ±2% in the range of 0.01 to 83 K lux. The indoor environment conditions were sampled every 5 minutes at an approximate height of 2.5 metres from ground level. The red letter “E” in Fig. 2 shows the location where the environmental measures took place.

### Procedures

Participants were recruited by promoting the art fair on social media. Before buying a ticket, participants were asked to provide informed consent and complete the online pre-questionnaire, see Fig. 1. Two links to the pre-questionnaire existed: one gave access to a free ticket and the other to a ticket that cost 5 euros. The content of both online pre-questionnaires was identical and we merged their responses. Each ticket was associated with a time slot during which visitors could enter the fair. At the fair, tickets were scanned, visitors walked by the cloakroom, and arrived at the research desk. At the research desk, we checked if visitors had filled in the pre-questionnaire using their email address. If not, e.g., because someone else bought their ticket, we only asked visitors to provide informed consent to participate in the study. To avoid congestion near the entrance, we did not ask these visitors to complete the pre-questionnaire on site. Next, an SDS attached to a key cord was handed out, and the visitor’s email address was registered to link the SDS and questionnaire data. If visitors came in groups, the linked tag IDs were registered. After registration, visitors activated their SDS at the activation desk, where they also received a small black bag to cover their sensor. We asked participants to activate their SDS themselves to avoid touching the disinfected materials. In the face mask condition, visitors were provided with a face mask and were asked to wear it until the end of the condition.

Once inside the fair, visitors could stay as long as desired. The maximum capacity inside was 150 visitors, which was never exceeded. Before exiting, visitors handed back the SDS, key cord, and bag at the back of the research desk, where their tag ID was once again registered. Finally, visitors were asked to scan a QR-code and complete the post-questionnaire. All materials were then disinfected, and the SDS charged before handed out again.

Between conditions, we performed two “data sweeps” by walking through the hall with the laptop and access point. The first collected all data from the sensors in the previous condition. The second activated the settings of the SDSs for the following condition. In addition, after the face mask condition, we informed visitors that they could take off their face mask; and when switching to or from a buzzer condition, we took in or handed out the black bags around the sensors. The camera and indoor environment measurements were performed during the entire art fair and did not require any interaction with visitors.

### Ethical issues

The University of Amsterdam collected the questionnaire and sensor data. The ethics review board of the University of Amsterdam (2020-CP-12488) approved data collection, and all participants provided informed consent before participating. The camera and indoor environment data were collected and processed by Centillien B.V., a Dutch company specialised in artificial intelligence and image recognition (https://centillien.com). Visitors were informed that the venue was filmed when they obtained a ticket. All personal identifiable information used to link the questionnaire and sensor data has been destroyed. The Ministry of Economic Affairs and Climate Policy (Ministerie van Economische Zaken en Klimaat, EZK) and Mondriaan fund invested in the production costs of the Art Fair and Smart Distance Lab organization.

## Data Records

The data set is available in comma-separated value (CSV) files on *figshare*^9^, and a relational (MySQL) database hosted by SURF SARA^10^. On *figshare*, the data are grouped based on the data source (i.e., questionnaire, sensor, camera, and environment). The database can be accessed via mysqlonubuntud.smartdist-uva.src.surf-hosted.nl/phpmyadmin, using the read-only account with username “sdl_guest” and password “dmebozY07tRfigfm”, or via any compatible analysis software or app. An example script to connect to the database via R can be found here: https://osf.io/2ag9z/. Table 2 provides an overview and short description of all data tables. Tables 3–7 describe each variable for each type of data table.

**Table 2:** Overview of data tables.

Group	Table	Description
	conditions	Overview conditions during art fair
camera	camera_codebook	Codebook
camera	camera_layer2_all	Unfiltered point detections of people from the raw footage
camera	camera_layer3_20200828_02_bidirectional_facemask_120	Layer 3 data of time slot 2 excluding entrance area, x between 0 and 845
camera	camera_layer3_20200828_03_bidirectional_nointervention_120	Layer 3 data of time slot 3 excluding entrance area, x between 0 and 845
camera	camera_layer3_20200828_04_bidirectional_buzzer_120	Layer 3 data of time slot 4 excluding entrance area, x between 0 and 845
camera	camera_layer3_20200829_06_unidirectional_nointervention_120	Layer 3 data of time slot 6 excluding entrance area, x between 0 and 845
camera	camera_layer3_20200829_07_unidirectional_buzzer_120	Layer 3 data of time slot 7 excluding entrance area, x between 0 and 845
camera	camera_layer3_20200830_09_nodirection_buzzer_120	Layer 3 data of time slot 9 excluding entrance area, x between 0 and 845
camera	camera_layer3_20200830_10_nodirection_buzzer_60	Layer 3 data of time slot 10 excluding entrance area, x between 0 and 845
camera	camera_layer3_20200830_11_nodirection_nointervention_60	Layer 3 data of time slot 11 excluding entrance area, x between 0 and 845
camera	camera_layer3_all	Filtered point detections of labeled individuals
camera	camera_map_all	Coordinates of walls in the art fair
camera	camera_map_codebook	Codebook
environment	environment_all	Temperature, humidity and light measures
environment	environment_codebook	Codebook
questionnaire	questionnaire_all	Responses to pre- and post-questionnaire
questionnaire	questionnaire_codebook	Codebook
sensor	sensor_all	SDS data of all conditions
sensor	sensor_codebook	Codebook
sensor	sensor_degree_all	Number of unique contacts per participant
sensor	sensor_degree_codebook	Codebook

All tables are available as.csv files on *figshare*^9^, and in a relational (MySQL) database^10^.

**Table 3:** Description of the sensor data.

Name	Description	Type	Values
timestamp	Timestamp of SDS data collected by access point in ISO 8601 format, timezone is CEST	String	YYYY-MM-DDTHH:mm:ss
day	Event day	Integer	1 = 2020-08-28
			2 = 2020-08-29
			3 = 2020-08-30
reporting_tagid	SDS tag id reporting contact	Integer	146–1176
reporting_qid	Unique person identifier wearing reporting SDS	Integer	11–998937
opposing_tagid	SDS tag id opposing contact	Integer	146–1176
opposing_qid	Unique person identifier wearing opposing SDS	Integer	11–998937
n_incidents	Number of contacts	Integer	0–224
timeslot	Time slot number	Integer	2–11
direction	Walking directions	Integer	0 = no directions
			1 = unidirectional
			2 = bidirectional
pre_q	Completed pre-questionnaire	Integer	0 = not completed
			1 = completed
post_q	Completed post-questionnaire	Integer	0 = not completed
			1 = completed
linked	SDS linked to household / group members	Integer	0 = not linked
			1 = at least 1 linked SDS
linked_id1	SDS tag id of first linked SDS	Integer	146–1176
linked_id2	SDS tag id of second linked SDS	Integer	146–1157
linked_id3	SDS tag id of third linked SDS, note no groups of 4	Integer	NA
location_tag	SDS has fixed location in art fair, note location tags do not have a QID	Integer	0 = SDS worn by visitor
			1 = fixed location SDS

QID is unique per person, while tag ID refers to the SDS and was handed out to multiple visitors during the art fair. A contact between two SDSs was registered on both sensors. Each sensor sent the data to the access point with their ID as reporting ID, and the other as opposing ID. When visitors from the same household linked their SDSs, their contacts were not registered.

**Table 4:** Description of the unique contacts data.

Name	Description	Type	Values
qid	Unique person identifier	Integer	11–998937
timeslot	Time slot number	Integer	2–11
degree	Number of unique contacts within timeslot	Integer	0–49

For each visitor, the number of unique contacts - within 1.5 metres - per time slot.

**Table 5:** Description of the camera data.

Name	Description	Type	Values
timestamp	Timestamp of detection in ISO 8601 microseconds format, timezone is CEST	String	YYYY-MM-DDTHH:mm:ss.ssssss
id	Unique person identifier, only in layer 3	Integer	0–29986
x	x coordinate in pixels, each pixel has a width and height of 5.5 cm	Integer	−99–1567
y	y coordinate in pixels, each pixel has a width and height of 5.5 cm	Integer	300–740

The “id” column is only present in the layer 3 tables, as detections have not been linked to unique visitors yet in layer 2. The full layer 2 and 3 tables contain all unfiltered and filtered detections of visitors respectively. The time slot specific tables of layer 3 only contain detections within the gallery area (0 ≤ x ≤ 845).

**Table 6:** Description of the map for the camera data.

Name	Description	Type	Values
wallid	Unique wall identifier	Integer	1–32
x1	x coordinate of left corner of wall	Integer	59–1240
y1	y coordinate of top corner of wall	Integer	394–626
x2	x coordinate of right corner of wall	Integer	62–1250
y2	y coordinate of bottom corner of wall	Integer	366–687

The map provides the coordinates of the 32 walls in the area of the art fair where camera data were collected.

**Table 7:** Description of the environmental data.

Name	Description	Type	Values
timestamp	Date and time of measurement in ISO 8601 format, timezone is CEST	String	YYYY-MM-DDTHH:mm:ss
temperature	Temperature in degrees Celsius	Numeric	19.7–28.3
humidity	Relative humidity	Integer	46–60
light	Light in lux	Numeric	0–626.9

Temperature, humidity, and light were measured every five minutes during the entire art fair.

## Technical Validation

When processing the data, both data checks and cleaning were conducted. The two pre-questionnaires were merged by email address. If participants completed a questionnaire multiple times, only the first completed questionnaire with a unique combination of email address, age, and gender was kept. A questionnaire ID (QID) was automatically assigned to participants who completed a questionnaire. Email addresses were replaced by their corresponding QID in both the questionnaire and sensor data to anonymise the data. We generated unique QIDs for participants in the sensor data who had not completed a questionnaire. We used QIDs instead of tag IDs to link the data sets since the SDSs were handed out multiple times a day to different visitors (see Fig. 1).

Contacts are stored twice in the sensor data, because a contact involves two SDSs. Both kept a record with their ID as reporting tag ID and the other as opposing tag ID. However, the sensor data also contained exact duplicates in the database, i.e., the same number of contacts with the same reporting and same opposing tag ID at the same time. In these cases, we only kept one of the records. We also removed a record if an SDS tag ID could not be linked to a QID of a person that was present at that time. These records could occur when an SDS was activated but not handed out, e.g., when removed from the charger and the SDS automatically activated. We added records to the sensor data when an SDS made zero contacts since the data should also include people without any contacts. These visitors were identified using the registration of tag IDs at the beginning and end of a visit. Finally, for each condition, we only kept the sensor data between the start and end data sweep of that condition.

**Fig. 3:**
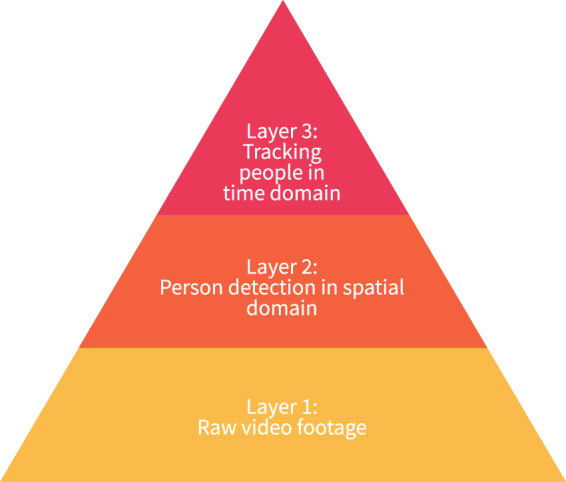
The layered structure of the camera data can be represented as a pyramid. Each layer depends on the previous layer and increases the abstraction of the data.

The camera data were processed and described in multiple layers, see Fig. 3. The layers follow a hierarchical structure such that each layer serves as an input to the next layer and increases the abstraction of the data^11^. Layer 1 contains the raw video footage captured during the art fair and is available upon request. Layer 2 provides the first level of abstraction from the raw video footage. Computer vision was used to obtain pixel coordinates of visitors. This abstraction was realised by using a sophisticated implementation of a blob detector, which included a K-nearest neighbour (KNN) background subtractor, morph dilation to reduce noise, and chain approximation. In this layer, the data of multiple cameras were merged into one single data set, and we removed data points where the camera views overlap. Each pixel has a width and height of 5.5 centimetres. The pixel coordinates obtained in both layer 2 and 3 can be converted to physical coordinates. The left upper corner of the map corresponds to the point where *x* = 0 and *y* = 0. Layer 3 adds a second level of abstraction by including time information to allow tracking of visitors over time. A centroid tracking algorithm in combination with filtering was used to provide data points where time gaps were reconstructed, and noise in the spatial-time domain was removed.

The indoor environment measures were taken at a height of 2.5 metres to prevent visitors from accessing the device.

## Usage Notes

For each table we provide a code book. We recommend reading the code book before accessing the data tables. To connect to the MySQL database, you might need the following information:

phpMyAdmin = mysqlonubuntud.smartdist-uva.src.surf-hosted.nl/phpmyadmin

user = sdl_guest

host = mysqlonubuntud.smartdist-uva.src.surf-hosted.nl

password = dmebozY07tRfigfm

db = sdl_202008_artfair

## Data Availability

All code related to this data set can be found in the Smart Distance Lab OSF project^12^.

## Online-only Table

**Online-only Table 1:** Questions and response options of pre- and post-questionnaires. Both the original Dutch and translated English versions are available.

Name	Description	Type	Values	Code	Original questions	Original response options
qid	Unique person identifier	Integer	12482–998937	qid		
pre_timestamp	Start date and time of pre-questionnaire in ISO 8601 format, timezone is CEST	String	YYYY-MM-DDTHH:mm:ss	timestamp		
completed	Which questionnaires were completed	Integer	1 = pre-questionnaire filled in 2 = pre- and post-questionnaire filled in 3 = post-questionnaire filled in	completed		
pre_duration_secs	Time in seconds to complete pre-questionnaire	Integer	83–346223	duration		
consent	I have read and understood the information, and …	Integer	1 = I agree to participate in the research and to the usage of the data 2 = I do not agree to participate in the study and the usage of the data NA = Participant gave consent on site, because pre-questionnaire was not completed	informed_consent	Ik heb de informatie gelezen en begrepen, en …	1 = Ik stem toe met deelname aan het onderzoek en gebruik van de daarmee verkregen gegevens 2 = Ik stem niet toe met deelname aan het onderzoek en gebruik van de daarmee verkregen gegevens
pre_q1	How old are you?	Integer	12–82	age	Hoe oud bent u?	
pre_q2	What is your gender?	Integer	1 = Male 2 = Female 3 = Other	gender	Wat is uw geslacht?	1 = Man 2 = Vrouw 3 = Anders
pre_q3	What is your highest completed education?	Integer	1 = Primary education, such as elementary school 2 = Secondary education, such as MAVO, HAVO, VWO, MBO 3 = Higher education, such as HBO or university 4 = Other/do not wish to disclose	education	Wat is uw hoogst afgeronde opleiding?	1 = Primair onderwijs, zoals basisschool 2 = Middelbaar onderwijs, zoals MAVO, HAVO, VWO, MBO 3 = Hoger onderwijs, zoals HBO of universitair 4 = Anders/wil ik niet zeggen
pre_q4	Are you or have you been infected with the coronavirus?	Integer	1 = Yes, confirmed by a test 2 = Yes, but not confirmed by a test 3 = No 4 = I don’t know	infected	Bent u besmet of besmet geweest met het coronavirus?	1 = Ja, bevestigd door een test 2 = Ja, maar niet bevestigd door een test 3 = Nee 4 = Weet ik niet
pre_q5	How likely do you think it is that you will be infected with the coronavirus next year?	Integer	0 = Very unlikely 100 = Very likely	infected_likelihood	Hoe waarschijnlijk denkt u dat het is dat u het aankomende jaar besmet raakt met het coronavirus?	0 = Zeer onwaarschijnlijk 100 = Zeer waarschijnlijk
pre_q6	How serious do you think a coronavirus infection would be for you?	Integer	1 = Not serious at all 7 = Very serious	infected_severity	Hoe ernstig denkt u dat een besmetting met het coronavirus voor u zou zijn?	1 = Totaal niet ernstig 7 = Zeer ernstig
pre_q7	For me personally, I estimate the health risk of an infection with the coronavirus as … large	Integer	1 = Extremely small 7 = Extremely large	health_risk_personal	Voor mij persoonlijk schat ik de gezondheidsrisico’s van een besmetting met het coronavirus als… groot	1 = Extreem klein 7 = Extreem groot
pre_q8	For my family and friends, I estimate the health risk of an infection with the coronavirus as … large	Integer	1 = Extremely small 7 = Extremely large	health_risk_close_others	Voor mijn familie en vrienden schat ik de gezondheidsrisico’s van een besmetting met het coronavirus als … groot	1 = Extreem klein 7 = Extreem groot
pre_q9	I worry about getting infected with the coronavirus myself	Integer	1 = No worries at all 7 = A lot of worries	worries_self	Ik maak me er zorgen over dat ik zelf besmet kan raken met het coronavirus	1 = Totaal geen zorgen 7 = Veel zorgen
pre_q10	I worry about infecting others with the coronavirus	Integer	1 = No worries at all 7 = A lot of worries	worries_others	Ik maak me er zorgen over dat ik anderen besmet met het coronavirus	1 = Totaal geen zorgen 7 = Veel zorgen
pre_q11	I worry that the health system will get overloaded	Integer	1 = No worries at all 7 = A lot of worries	system_overloaded	Ik maak me zorgen dat het gezondheidssysteem overbelast wordt	1 = Totaal geen zorgen 7 = Veel zorgen
pre_q12	I think that most individuals keep 1.5 meters distance as much as possible	Integer	1 = Completely disagree 7 = Completely agree	sd_others	Ik denk dat de meeste mensen zoveel mogelijk 1.5 meter afstand houden	1 = Helemaal mee oneens 7 = Helemaal mee eens
pre_q13	I think that most individuals think it is important to keep 1.5 meters distance as much as possible	Integer	1 = Completely disagree 7 = Completely agree	sd_importance_others	Ik denk dat de meeste mensen het belangrijk vinden dat men zoveel mogelijk 1.5 meter afstand houdt	1 = Helemaal mee oneens 7 = Helemaal mee eens
pre_q14	I experience the rule of keeping 1.5 meters distance as much as possible as … sensible	Integer	1 = Not sensible 7 = Sensible	sd_sensible	Ik ervaar de regel om zoveel mogelijk 1.5 meter afstand te houden als … verstandig	1 = Onverstandig 7 = Verstandig
pre_q15	I experience the rule of keeping 1.5 meters distance as much as possible as … usefull	Integer	1 = Useless 7 = Usefull	sd_useful	Ik ervaar de regel om zoveel mogelijk 1.5 meter afstand te houden als … nuttig	1 = Nutteloos 7 = Nuttig
pre_q16	I experience the rule of keeping 1.5 meters distance as much as possible as … enjoyable	Integer	1 = Not enjoyable 7 = Enjoyable	sd_enjoyable	Ik ervaar de regel om zoveel mogelijk 1.5 meter afstand te houden als … prettig	1 = Onprettig 7 = Prettig
pre_q17	I experience the rule of keeping 1.5 meters distance as much as possible as … fair	Integer	1 = Unfair 7 = Fair	sd_fair	Ik ervaar de regel om zoveel mogelijk 1.5 meter afstand te houden als … eerlijk	1 = Oneerlijk 7 = Eerlijk
pre_q18	I experience the rule of keeping 1.5 meters distance as much as possible as … acceptable	Integer	1 = Inacceptable 7 = Acceptable	sd_acceptable	Ik ervaar de regel om zoveel mogelijk 1.5 meter afstand te houden als … acceptabel	1 = Onacceptabel 7 = Acceptabel
pre_q19	I experience the rule of keeping 1.5 meters distance as much as possible as … easy	Integer	1 = Difficult 7 = Easy	sd_easy	Ik ervaar de regel om zoveel mogelijk 1.5 meter afstand te houden als … makkelijk	1 = Moeilijk 7 = Makkelijk
pre_q20	I think that keeping 1.5 meters distance helps to prevent het spread of the coronavirus	Integer	1 = Completely disagree 7 = Completely agree	sd_helps	Ik denk dat het houden van 1.5 m aftstand helpt de verspreiding van het coronavirus te voorkomen	1 = Helemaal mee oneens 7 = Helemaal mee eens
pre_q21	In our society, we have to keep 1.5 meters distance to prevent the spread of the coronavirus	Integer	1 = Completely disagree 7 = Completely agree	sd_necessary	In onze maatschappij moeten we 1.5 m afstand houden om de verspreiding van het coronavirus te voorkomen	1 = Helemaal mee oneens 7 = Helemaal mee eens
pre_q22	I know what I have to do to keep 1.5 meters distance to others	Integer	1 = Completely disagree 7 = Completely agree	sd_knowledge	Ik weet wat ik moet doen om 1.5 meter afstand te houden tot anderen	1 = Helemaal mee oneens 7 = Helemaal mee eens
pre_q23	I adhere to the 1.5 meters rule	Integer	1 = I never adhere to the rule 7 = I always adhere to the rule	sd_adherence	Ik houd me aan de 1.5 meter regel	1 = Ik volg de maatregelen nooit 7 = Ik volg de maatregelen altijd
pre_q24	Keeping 1.5 meters distance in public spaces is something … that I do automatically	Integer	1 = Completely disagree 7 = Completely agree	sd_automatic	1.5 meter afstand houden in de publieke ruimte is iets… wat ik automatisch doe	1 = Helemaal mee oneens 7 = Helemaal mee eens
pre_q25	Keeping 1.5 meters distance in public spaces is something … that I do without thinking about it	Integer	1 = Completely disagree 7 = Completely agree	sd_naturally	1.5 meter afstand houden in de publieke ruimte is iets… wat ik doe zonder er over na te denken	1 = Helemaal mee oneens 7 = Helemaal mee eens
pre_q26	Keeping 1.5 meters distance in public spaces is something … that I start doing before I realize that I do it	Integer	1 = Completely disagree 7 = Completely agree	sd_prerealize	1.5 meter afstand houden in de publieke ruimte is iets… wat ik begin te doen voordat ik mij realiseer dat ik het doe	1 = Helemaal mee oneens 7 = Helemaal mee eens
pre_q27	Keeping 1.5 meters distance in public spaces is something … that I do not have to consciously remember	Integer	1 = Completely disagree 7 = Completely agree	sd_unconcious	1.5 meter afstand houden in de publieke ruimte is iets… wat ik niet bewust hoef te onthouden	1 = Helemaal mee oneens 7 = Helemaal mee eens
pre_q28	I think that facemasks are a good protection against infections with the coronavirus	Integer	1 = Completely disagree 7 = Completely agree	facemask_protect	Ik denk dat mondkapjes een goede bescherming bieden tegen besmetting met het coronavirus	1 = Helemaal mee oneens 7 = Helemaal mee eens
pre_q29	I think that keeping 1.5 meters distance is less important when wearing a facemask	Integer	1 = Completely disagree 7 = Completely agree	facemask_less_sd	Ik denk dat door het dragen van een mondkapje het houden van 1.5 meter afstand minder belangrijk is	1 = Helemaal mee oneens 7 = Helemaal mee eens
pre_q30	How is your health in general?	Integer	1 = Very bad 7 = Very good	health	Hoe is over het algemeen uw gezondheid?	1 = Zeer slecht 7 = Zeer goed
post_timestamp	Start date and time of post-questionnaire in ISO 8601 format, timezone is CEST	String	YYYY-MM-DDTHH:mm:ss	timestamp		
post_duration_secs	Time in seconds to complete post-questionnaire	Integer	51–6986	duration		
post_q1	I have tried to keep 1.5 meters distance	Integer	1 = Completely disagree 7 = Completely agree	sd_try	Ik heb geprobeerd om 1.5 meter afstand te houden	1 = Helemaal mee oneens 7 = Helemaal mee eens
post_q2	Assessing when someone is within 1.5 meters distance from me, I experienced as … easy	Integer	1 = Difficult 7 = Easy	sd_assess	Bepalen wanneer iemand binnen 1.5 m afstand van mij komt heb ik ervaren als … makkelijk	1 = Moeilijk 7 = Makkelijk
post_q3	I have experienced keeping 1.5 meters distance in this room as … easy	Integer	1 = Difficult 7 = Easy	sd_possible	Ik heb het 1.5 meter afstand houden in deze ruimte ervaren als … makkelijk	1 = Moeilijk 7 = Makkelijk
post_q4	I adhered to the 1.5 meters distance rule	Integer	1 = Not at all 7 = Constantly	sd_adhere	Ik heb mij gehouden aan de 1.5 meter afstand regel	1 = Helemaal niet 7 = Voortdurend
post_q5	Keeping 1.5 meters distance in this room is something that I did automatically	Integer	1 = Completely disagree 7 = Completely agree	sd_automatic_post	1.5 meter afstand houden in deze ruimte is iets dat ik automatisch deed	1 = Helemaal mee oneens 7 = Helemaal mee eens
post_q6	I was constantly reminded to keep 1.5 meters distance	Integer	1 = Completely disagree 7 = Completely agree	reminded	Ik werd voortdurend herinnerd aan het houden van 1.5 meter afstand	1 = Helemaal mee oneens 7 = Helemaal mee eens
post_q7	I felt protected against infections with the coronavirus	Integer	1 = Completely disagree 7 = Completely agree	protected	Ik voelde me beschermd tegen besmetting met het coronavirus	1 = Helemaal mee oneens 7 = Helemaal mee eens
post_q8	I found it stressful to be in this room	Integer	1 = Completely disagree 7 = Completely agree	stressful	Ik vond het stressvol om in deze ruimte aanwezig te zijn	1 = Helemaal mee oneens 7 = Helemaal mee eens
post_q9	I had the feeling that I had a choice and freedom in the things I did	Integer	1 = Completely disagree 7 = Completely agree	freedom	Ik had het gevoel dat ik keuze en vrijheid had in de dingen die ik deed	1 = Helemaal mee oneens 7 = Helemaal mee eens
post_q10	The things I did felt as if I had to do them	Integer	1 = Completely disagree 7 = Completely agree	mandatory	De dingen die ik deed voelde aan alsof ze moesten	1 = Helemaal mee oneens 7 = Helemaal mee eens
post_q11	I felt obliged to do a lot of things that I would not choose myself	Integer	1 = Completely disagree 7 = Completely agree	obliged	Ik voelde mij gedwongen veel dingen te doen waar ik zelf niet voor zou kiezen	1 = Helemaal mee oneens 7 = Helemaal mee eens
post_q12	I trusted that I could keep 1.5 meters distance well	Integer	1 = Completely disagree 7 = Completely agree	sd_trust	Ik vertrouwde erop dat ik goed 1.5 m afstand kon bewaren	1 = Helemaal mee oneens 7 = Helemaal mee eens
post_q13	I have experienced my visit as pleasant	Integer	1 = Completely disagree 7 = Completely agree	pleasure	Ik heb het bezoek als plezierig ervaren	1 = Helemaal mee oneens 7 = Helemaal mee eens
post_q14	How many glasses of alcohol did you drink during your visit?	Integer	1 = I did not drink alcohol 2 = 1 glass 3 = 2 glasses 4 = 3 glasses 5 = 4 glasses 6 = 5 glasses 7 = 6 glasses or more	alcohol	Hoeveel glazen alcohol heeft u tijdens uw bezoek gedronken?	1 = Ik heb geen alcohol gedronken 2 = 1 glas 3 = 2 glazen 4 = 3 glazen 5 = 4 glazen 6 = 5 glazen 7 = 6 glazen of meer
post_q15	Did you wear a facemask during your visit?	Integer	1 = No 2 = Yes	facemask	Heeft u tijdens uw bezoek een mondkapje gedragen?	1 = Nee 2 = Ja

## References

[1] van Leeuwen M, Klerks Y, Bargeman B, Heslinga J, Bastiaansen M (2020). Leisure will not be locked down - insights on leisure and COVID-19 from the Netherlands. World Leisure Journal.

[2] Rijksoverheid. Corona en regels voor afstand houden. https://www.rijksoverheid.nl/onderwerpen/coronavirus-covid-19/openbaar-en-dagelijks-leven/afstand-houden (2020).

[3] Dong E, Du H, Gardner L (2020). An interactive web-based dashboard to track COVID-19 in real time. The Lancet Infectious Diseases.

[4] Borsboom, D. *et al*. The lighting of the BECONs: A behavioral data science approach to tracking interventions in COVID-19 research. Preprint at https://psyarxiv.com/53ey9 (2020).

[5] Smart Distance Lab. Bevindingen. https://smartdistancelab.nl/bevindingen-2/ (2020).

[6] Blanken, T. F. *et al*. The Smart Distance Lab: A new methodology for assessing social distancing interventions. Preprint at https://osf.io/mjg2f (2020).

[7] Gardner B, Abraham C, Lally P, de Bruijn GJ (2012). Towards parsimony in habit measurement: Testing the convergent and predictive validity of an automaticity subscale of the Self-Report Habit Index. International Journal of Behavioral Nutrition and Physical Activity.

[8] UbiBot. UbiBot WS1 User Guide. https://www.ubibot.com/wp-content/uploads/dlm_uploads/2020/12/WS1-User-Guide-English.pdf (2020).

[9] Tanis CC (2021). figshare.

[10] Tanis, C. C. *et al*. Smart Distance Lab: Relational MySQL Database. http://sdl.smartdist-uva.surf-hosted.nl/ (2021).

[11] Knoppers, J., Markus, D. A. W. & Kanters, G. Fever detection, distance measurement, headcount and group formation analysis using computer vision software Intra to detect SARS-CoV-2 regulation violations. https://centillien.com/wp-content/uploads/2020/11/Whitepaper-Covid-19-measurements.pdf (2020).

[12] Blanken TF, Tanis CC, Borsboom D, van Harreveld F (2021). OSF.

